# Supplementary figures and images for: Quantitative plasma proteomics identifies metallothioneins as a marker of acute-on-chronic liver failure associated acute kidney injury
Source: Front Immunol. 2023 Jan 26;13:1041230. doi: 10.3389/fimmu.2022.1041230 (PMC9909472; doi:10.3389/fimmu.2022.1041230)

## Slide 1
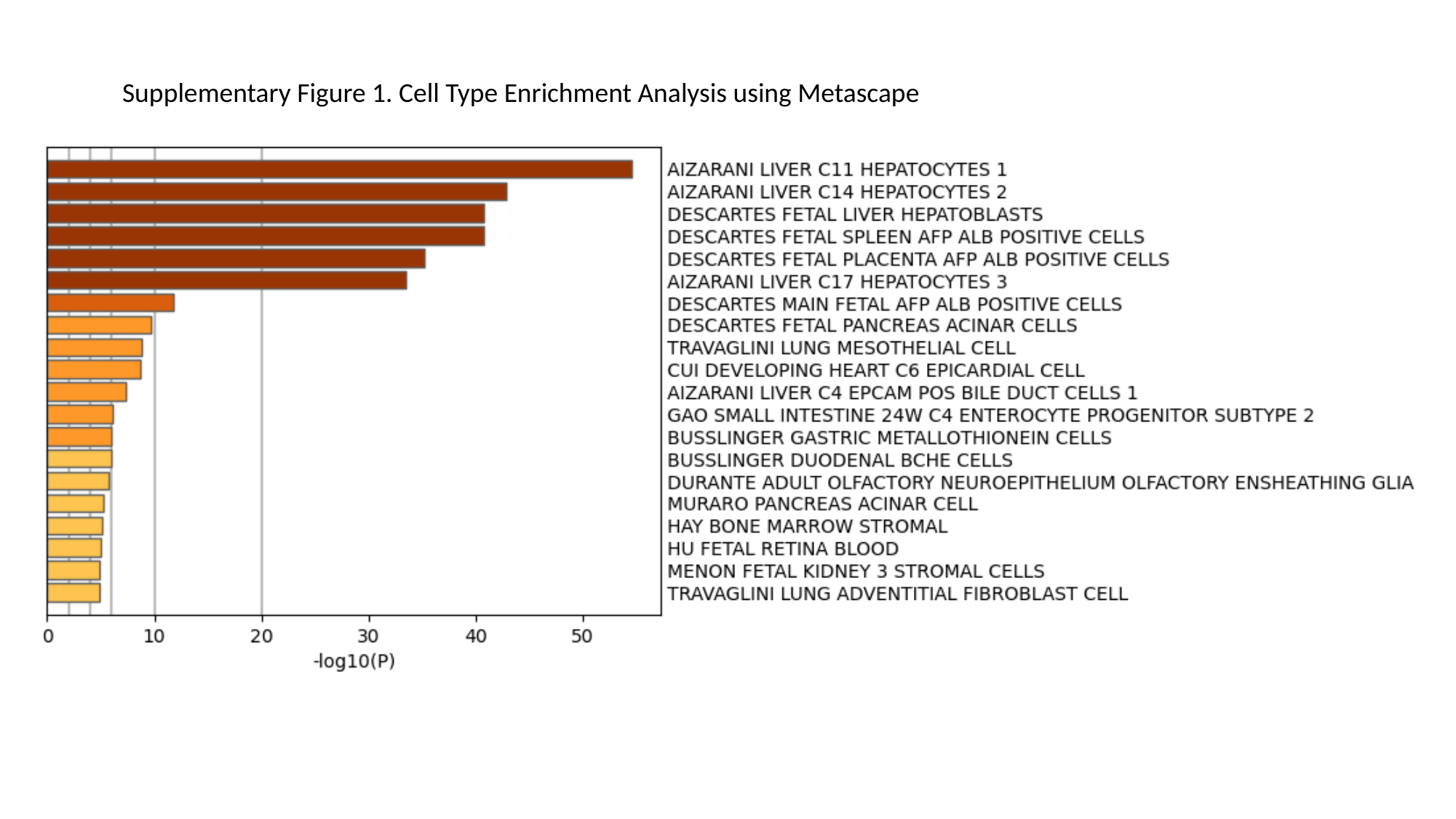

Supplementary Figure 1. Cell Type Enrichment Analysis using Metascape

Supplement: Supplementary file 1 [file Presentation_1.pptx]
