# Supplementary material for: Quantitative plasma proteomics identifies metallothioneins as a marker of acute-on-chronic liver failure associated acute kidney injury
Source: Front Immunol. 2023 Jan 26;13:1041230. doi: 10.3389/fimmu.2022.1041230 (PMC9909472; doi:10.3389/fimmu.2022.1041230)
Supplement: Supplementary file 2 [file Presentation_2.pptx]

## Slide 1
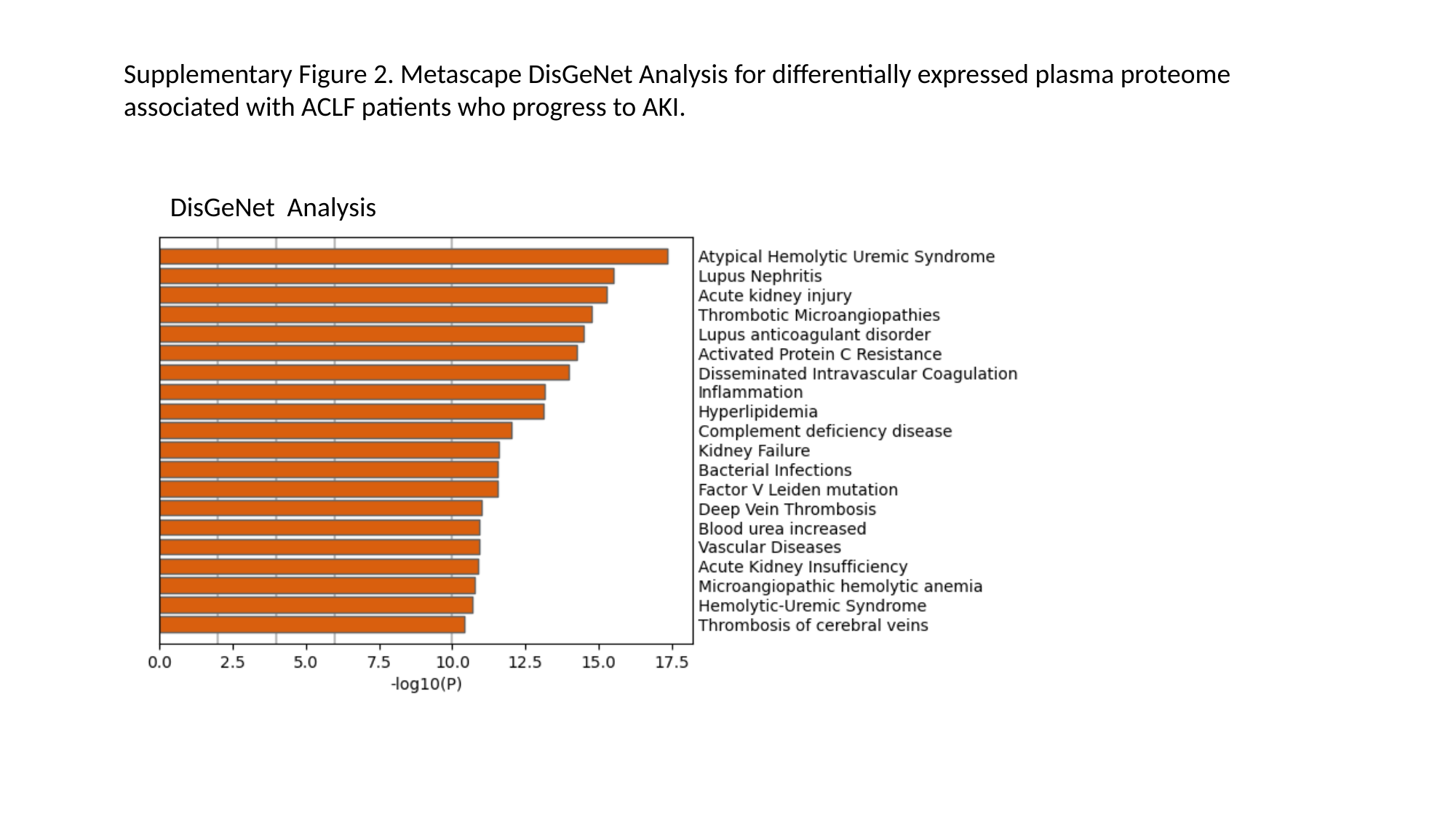

Supplementary Figure 2. Metascape DisGeNet Analysis for differentially expressed plasma proteome associated with ACLF patients who progress to AKI.
DisGeNet Analysis
