# Supplementary material for: Quantitative plasma proteomics identifies metallothioneins as a marker of acute-on-chronic liver failure associated acute kidney injury
Source: Front Immunol. 2023 Jan 26;13:1041230. doi: 10.3389/fimmu.2022.1041230 (PMC9909472; doi:10.3389/fimmu.2022.1041230)
Supplement: Supplementary file 4 [file Presentation_4.pptx]

## Slide 1
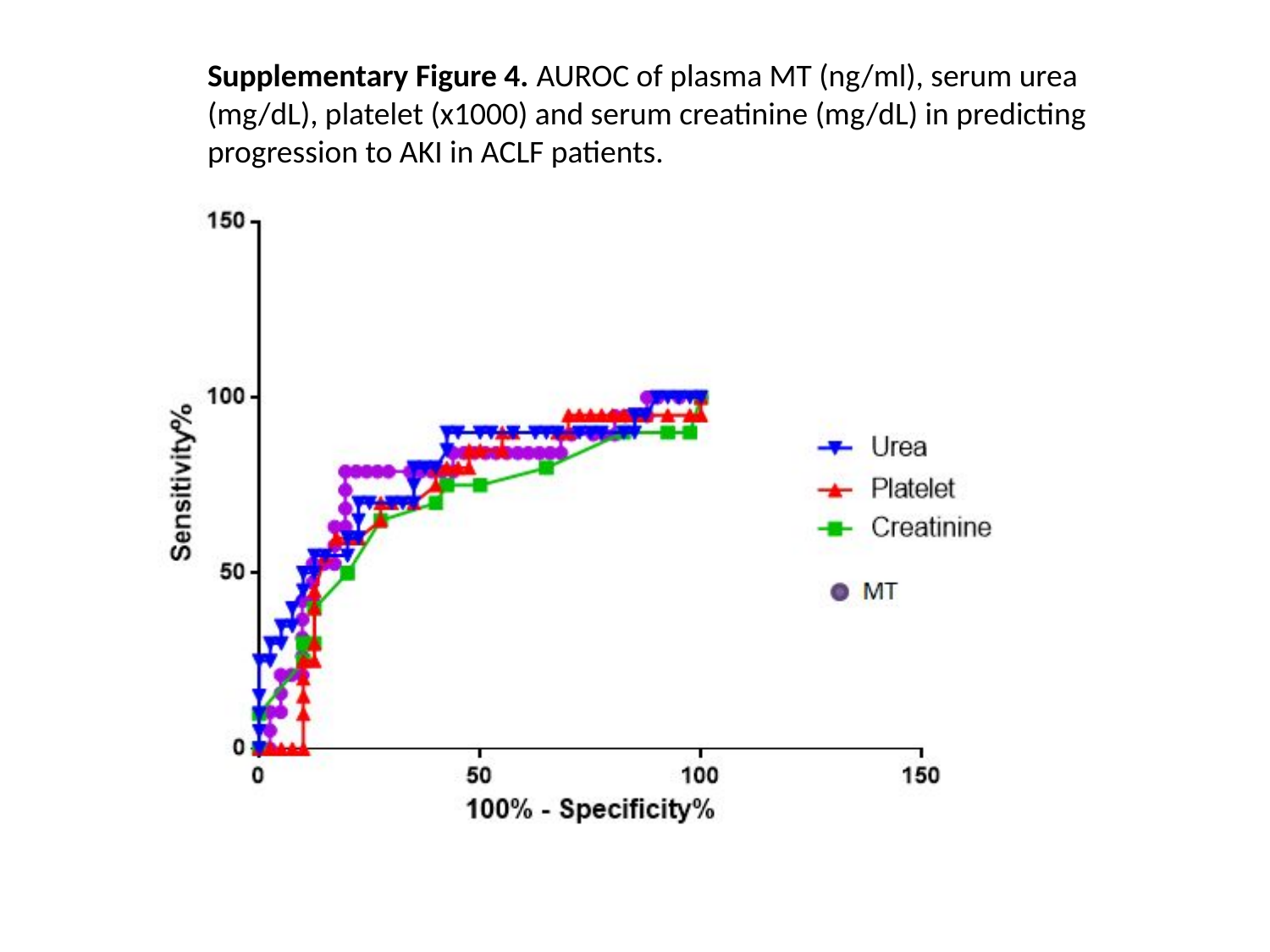

Supplementary Figure 4. AUROC of plasma MT (ng/ml), serum urea (mg/dL), platelet (x1000) and serum creatinine (mg/dL) in predicting progression to AKI in ACLF patients.
