# Supplementary material for: Quantitative plasma proteomics identifies metallothioneins as a marker of acute-on-chronic liver failure associated acute kidney injury
Source: Front Immunol. 2023 Jan 26;13:1041230. doi: 10.3389/fimmu.2022.1041230 (PMC9909472; doi:10.3389/fimmu.2022.1041230)
Supplement: Supplementary file 5 [file Presentation_5.pptx]

## Slide 1
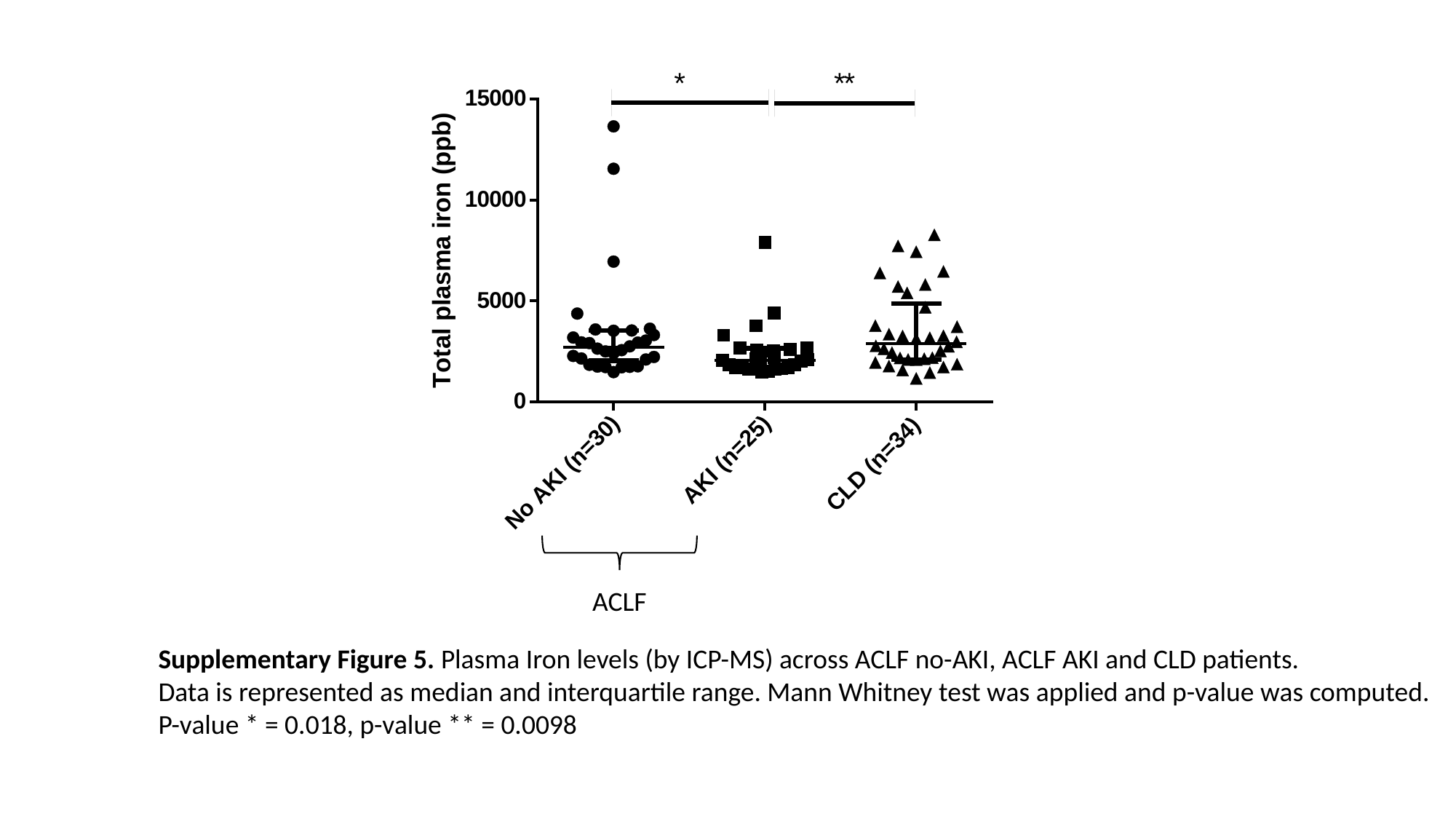

ACLF
Supplementary Figure 5. Plasma Iron levels (by ICP-MS) across ACLF no-AKI, ACLF AKI and CLD patients.
Data is represented as median and interquartile range. Mann Whitney test was applied and p-value was computed.
P-value * = 0.018, p-value ** = 0.0098
