# Supplementary material for: Quantitative plasma proteomics identifies metallothioneins as a marker of acute-on-chronic liver failure associated acute kidney injury
Source: Front Immunol. 2023 Jan 26;13:1041230. doi: 10.3389/fimmu.2022.1041230 (PMC9909472; doi:10.3389/fimmu.2022.1041230)
Supplement: Supplementary file 6 [file Presentation_6.pptx]

## Slide 1
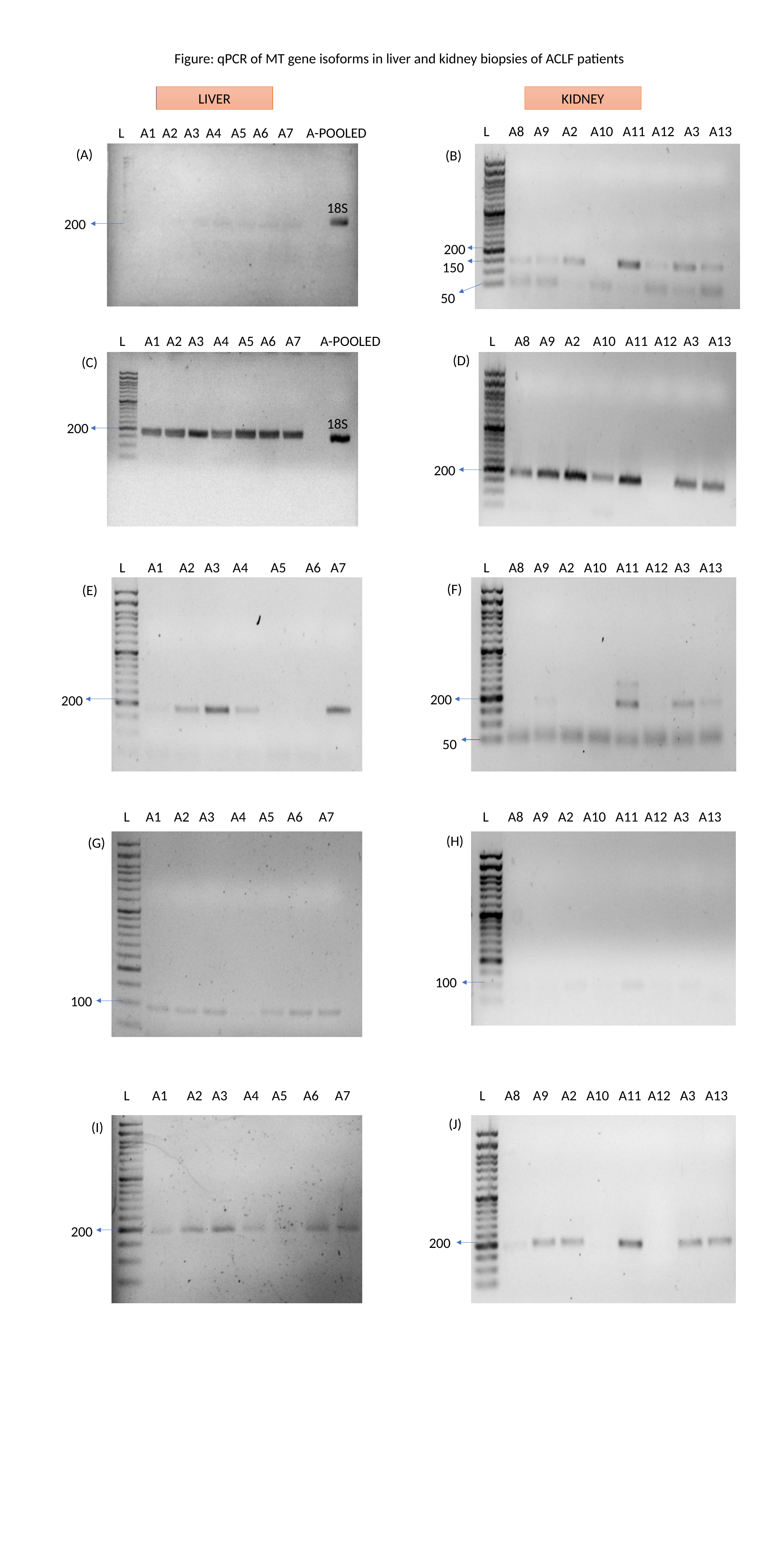

Figure: qPCR of MT gene isoforms in liver and kidney biopsies of ACLF patients
LIVER
KIDNEY
 L A8 A9 A2 A10 A11 A12 A3 A13
 L A1 A2 A3 A4 A5 A6 A7 A-POOLED
(A)
(B)
18S
200
200
150
50
 L A1 A2 A3 A4 A5 A6 A7 A-POOLED
 L A8 A9 A2 A10 A11 A12 A3 A13
(D)
(C)
18S
200
200
 L A1 A2 A3 A4 A5 A6 A7
 L A8 A9 A2 A10 A11 A12 A3 A13
(F)
(E)
200
200
50
 L A1 A2 A3 A4 A5 A6 A7
 L A8 A9 A2 A10 A11 A12 A3 A13
(H)
(G)
100
100
 L A1 A2 A3 A4 A5 A6 A7
 L A8 A9 A2 A10 A11 A12 A3 A13
(J)
(I)
200
200

## Slide 2
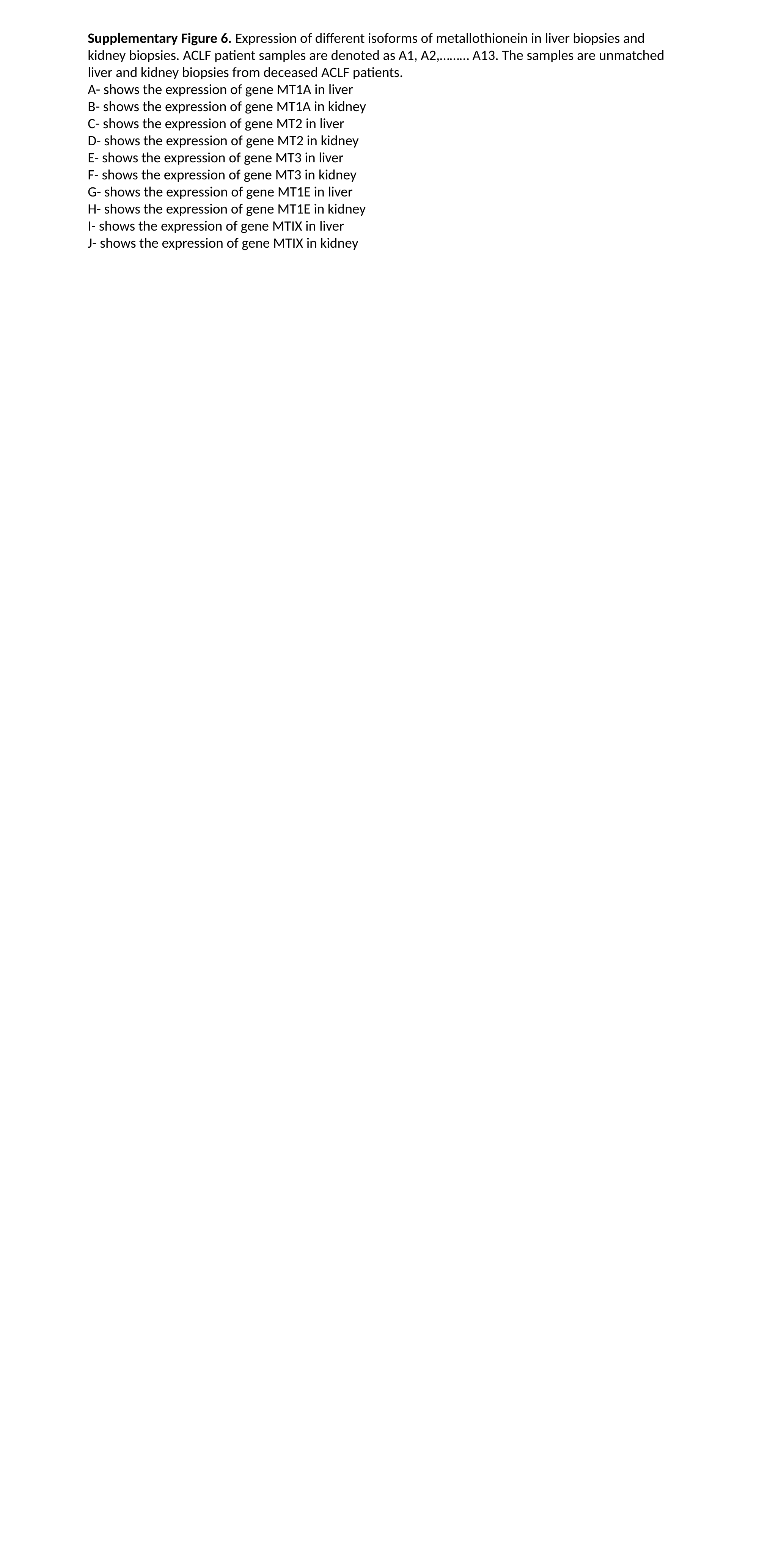

Supplementary Figure 6. Expression of different isoforms of metallothionein in liver biopsies and kidney biopsies. ACLF patient samples are denoted as A1, A2,……… A13. The samples are unmatched liver and kidney biopsies from deceased ACLF patients.
A- shows the expression of gene MT1A in liver
B- shows the expression of gene MT1A in kidney
C- shows the expression of gene MT2 in liver
D- shows the expression of gene MT2 in kidney
E- shows the expression of gene MT3 in liver
F- shows the expression of gene MT3 in kidney
G- shows the expression of gene MT1E in liver
H- shows the expression of gene MT1E in kidney
I- shows the expression of gene MTIX in liver
J- shows the expression of gene MTIX in kidney
