# Supplementary material for: Quantitative plasma proteomics identifies metallothioneins as a marker of acute-on-chronic liver failure associated acute kidney injury
Source: Front Immunol. 2023 Jan 26;13:1041230. doi: 10.3389/fimmu.2022.1041230 (PMC9909472; doi:10.3389/fimmu.2022.1041230)
Supplement: Supplementary file 7 [file Presentation_7.pptx]

## Slide 1
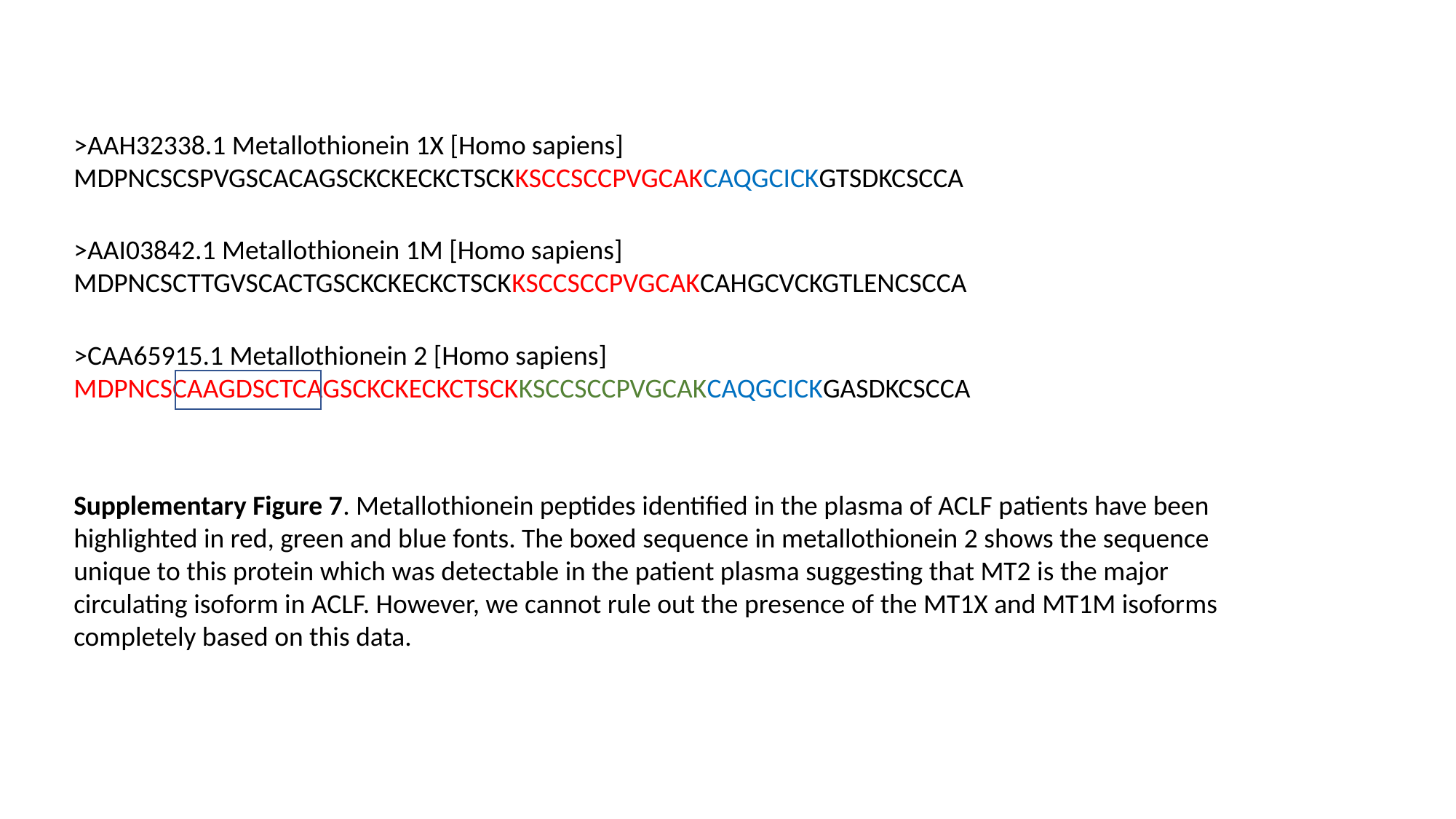

>AAH32338.1 Metallothionein 1X [Homo sapiens] MDPNCSCSPVGSCACAGSCKCKECKCTSCKKSCCSCCPVGCAKCAQGCICKGTSDKCSCCA
>AAI03842.1 Metallothionein 1M [Homo sapiens] MDPNCSCTTGVSCACTGSCKCKECKCTSCKKSCCSCCPVGCAKCAHGCVCKGTLENCSCCA
>CAA65915.1 Metallothionein 2 [Homo sapiens] MDPNCSCAAGDSCTCAGSCKCKECKCTSCKKSCCSCCPVGCAKCAQGCICKGASDKCSCCA
Supplementary Figure 7. Metallothionein peptides identified in the plasma of ACLF patients have been highlighted in red, green and blue fonts. The boxed sequence in metallothionein 2 shows the sequence unique to this protein which was detectable in the patient plasma suggesting that MT2 is the major circulating isoform in ACLF. However, we cannot rule out the presence of the MT1X and MT1M isoforms completely based on this data.
