# Supplementary material for: Quantitative plasma proteomics identifies metallothioneins as a marker of acute-on-chronic liver failure associated acute kidney injury
Source: Front Immunol. 2023 Jan 26;13:1041230. doi: 10.3389/fimmu.2022.1041230 (PMC9909472; doi:10.3389/fimmu.2022.1041230)
Supplement: Supplementary file 9 [file Table_2.docx]

**Supplementary Table 2.** **Correlation matrix for plasma MT (ng/ml) v/s blood parameters and eGFR.**

Plasma metallothionein (MT) levels of ACLF patients (n=93, both AKI and no AKI combined) were compared with blood parameters and estimated glomerular filtration rate (eGFR) (calculated by CKD-EPI formula) and a correlation matrix was generated in GraphPad Prism. A pair-wise Spearman correlation coefficient was calculated and p-value significance was computed.

| MT (ng/ml) | Spearman’s rank correlation coefficient | P-value |
| --- | --- | --- |
| v/s |  |  |
| Total leucocyte count (TLC) (per mm3) | 0.296 | 0.004 |
| Platelet x 1000 | 0.068 | 0.518 |
| Urea (mg/dL) | 0.359 | < 0.0001 |
| Creatinine (mg/dL) | 0.412 | < 0.0001 |
| Bilirubin (mg/dL) | -0.022 | 0.831 |
| AST (IU/L) | 0.113 | 0.282 |
| ALT (IU/L) | 0.046 | 0.663 |
| SAP (IU/L) | 0.243 | 0.019 |
| Albumin (g/dL) | 0.006 | 0.955 |
| eGFR (mL/min per 1.73m^2^) | -0.359 | <0.0001 |
