# Supplementary material for: Quantitative plasma proteomics identifies metallothioneins as a marker of acute-on-chronic liver failure associated acute kidney injury
Source: Front Immunol. 2023 Jan 26;13:1041230. doi: 10.3389/fimmu.2022.1041230 (PMC9909472; doi:10.3389/fimmu.2022.1041230)
Supplement: Supplementary file 10 [file Table_3.docx]

|  |  | Correlation coefficient | P-value |
| --- | --- | --- | --- |
| ACLF no-AKI | Hb (g/dL) | 0.315 | 0.090 |
|  | PLT (x1000) | 0.132 | 0.485 |
|  | Urea (mg/dL) | -0.095 | 0.617 |
|  | Creatinine (mg/dL) | 0.088 | 0.644 |
|  | Bilirubin (mg/dL) | 0.233 | 0.215 |
|  | AST (IU/L) | 0.139 | 0.465 |
|  | ALT (IU/L) | 0.194 | 0.305 |
|  | SAP (IU/L) | 0.092 | 0.627 |
|  | Albumin (g/dL) | 0.137 | 0.471 |
|  |  |  |  |
| ACLF AKI | Hb (g/dL) | 0.253 | 0.222 |
|  | PLT (x1000) | 0.214 | 0.304 |
|  | Urea (mg/dL) | -0.083 | 0.693 |
|  | Creatinine (mg/dL) | -0.058 | 0.782 |
|  | Bilirubin (mg/dL) | 0.306 | 0.136 |
|  | AST (IU/L) | 0.015 | 0.945 |
|  | ALT (IU/L) | -0.117 | 0.579 |
|  | SAP (IU/L) | 0.002 | 0.994 |
|  | Albumin (g/dL) | 0.191 | 0.361 |
|  |  |  |  |

**Supplementary Table 3. Correlation of plasma Fe (ICP-MS) with blood parameters in ACLF no-AKI and ACLF AKI**
